# Molar tooth carbonates and benthic methane fluxes in Proterozoic oceans

**DOI:** 10.1038/ncomms10317

**Published:** 2016-01-07

**Authors:** Bing Shen, Lin Dong, Shuhai Xiao, Xianguo Lang, Kangjun Huang, Yongbo Peng, Chuanming Zhou, Shan Ke, Pengju Liu

**Affiliations:** 1 Key Laboratory of Orogenic Belts and Crustal Evolution, MOE, Beijing 100871, China; 2 School of Earth and Space Sciences, Peking University, No.5 Yiheyuan Road Haidian District, Beijing 100871, China; 3 Department of Geosciences, Virginia Tech, Blacksburg, Virginia 24061, USA; 4 Department of Geology and Geophysics, Louisiana State University, Baton Rouge, Louisiana 70803, USA; 5 Key Laboratory of Economic Stratigraphy and Palaeogeography, Nanjing Institute of Geology and Palaeontology, Chinese Academy of Sciences, Nanjing 210008, China; 6 State Key Laboratory of Geological Processes and Mineral Resources, China University of Geosciences, Beijing 100083, China; 7 Institute of Geology, Chinese Academy of Geological Sciences, Beijing 100037, China

## Abstract

Molar tooth structures are ptygmatically folded and microspar-filled structures common in early- and mid-Proterozoic (∼2,500–750 million years ago, Ma) subtidal successions, but extremely rare in rocks <750 Ma. Here, on the basis of Mg and S isotopes, we show that molar tooth structures may have formed within sediments where microbial sulphate reduction and methanogenesis converged. The convergence was driven by the abundant production of methyl sulphides (dimethyl sulphide and methanethiol) in euxinic or H_2_S-rich seawaters that were widespread in Proterozoic continental margins. In this convergence zone, methyl sulphides served as a non-competitive substrate supporting methane generation and methanethiol inhibited anaerobic oxidation of methane, resulting in the buildup of CH_4_, formation of degassing cracks in sediments and an increase in the benthic methane flux from sediments. Precipitation of crack-filling microspar was driven by methanogenesis-related alkalinity accumulation. Deep ocean ventilation and oxygenation around 750 Ma brought molar tooth structures to an end.

## Abstract (web summary)

Molar tooth structures are common in early- to mid-Proterozoic carbonates but extremely rare in rocks younger than 750 Ma. Here, the authors show molar tooth carbonate formation is related to benthic methane fluxes.

## Graphical abstract

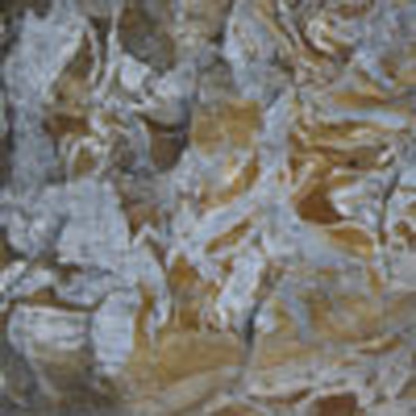

Molar tooth carbonates (MTCs), or carbonate rocks containing molar tooth structures (MTSs), occur mostly if not exclusively in successions deposited in subtidal environments before 750 Ma (refs 1, 2). The formation of MTCs requires the generation of cracks within unconsolidated sediments, followed by the rapid infilling of such cracks with early diagenetic calcispar before sediment compaction. The formation of molar tooth (MT) cracks have been variously related to subaqueous syneresis^3^, gas bubble expansion resulting from CH_4_, H_2_S or CO_2_ degassing^2^,^4^,^5^,^6^ and seismic activities^7^,^8^. The disappearance of MTCs at around 750 Ma has been related to the rise of animals^5^,^7^,^8^,^9^, a drop in calcite saturation of seawater^1^,^2^,^10^ or an increase in the concentrations of calcite precipitation inhibitors such as Fe^2+^, Mg^2+^, SO_4_^2–^ or PO_4_ (refs 1, 2, 3, 11).

To illuminate the origin of MTCs, we measured the Mg, S and C isotopic compositions of MTCs from the early Neoproterozoic (1,000–750 Ma) Wanlong Formation in southern Jilin Province of North China (Supplementary Fig. 1). In the Wanlong Formation, MTSs are abundant within the thick-bedded argillaceous lime mudstone that is intercalated with the finely laminated limestone (Supplementary Note 1 and Supplementary Figs 2 and 3a). Sedimentological evidence, including the predominance of parallel bedding and the lack of subaerial exposure structures, indicates that MTCs in the Wanlong Formation was deposited below fair-weather wave base^12^. S isotopic data indicate that MT microspar was precipitated within microbial sulphate reduction (MSR) zone and Mg isotopic data suggest that microspar precipitation predated the dolomitization of host rock. We propose that MT microspar was precipitated in the sediment column where MSR and methanogenesis occur simultaneously underneath sulphidic seawaters and where the production of CH_4_ from methyl sulphides and the inhibition of CH_4_ oxidation by methanethiol allowed CH_4_ to build up in the sediments.

## Results

### Petrographic observations of the MTCs

MTSs are normally oriented vertically or obliquely with respect to bedding planes and show clear cross-cutting relationships with each other (Supplementary Fig. 3b–d). MT cracks are filled with microcrystalline calcite crystals (MT microspars) ranging from 10 to 20 μm in size (Supplementary Fig. 3e,f). The argillaceous host rocks (with an average siliciclastic content of 33.4 wt%, Supplementary Table 5) are partially dolomitized (Supplementary Fig. 3g,h).

### Isotopic compositions of the MTCs

**Figure 1:**
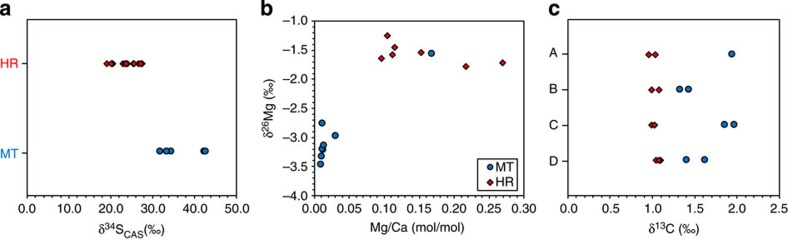
Isotopic compositions of MTCs. (**a**) S isotopic compositions of CAS from MT calcispars (MT) and host rocks (HR). (**b**) Cross-plot of Mg/Ca (molar ratio) versus *δ*^26^Mg. The argillaceous host rock has higher Mg/Ca ratios and is enriched in ^26^Mg than MT calcispars. (**c**) C isotopic compositions of MT calcispars (MT) versus host rock (HR) of four samples (A, B, C and D).

Sulphur isotopic values of carbonate-associated sulphate (CAS) extracted from MT microspars (*δ*^34^S_MT_: 31.9–42.8‰) are higher than those of CAS from calcareous host rock (*δ*^34^S_HR_: 19.1–27.6‰; Fig. 1a, Supplementary Fig. 4 and Supplementary Table 2). Mg isotopic compositions of MT microspars (*δ*^26^Mg_MT_) is around –3.3‰ (relative to DSM3), ∼1.6‰ lower than those of the host rock (*δ*^26^Mg_HR_; Fig. 1b, Supplementary Fig. 5 and Supplementary Table 1). C isotopes of MT microspars (*δ*^13^C_MT_) are systematically heavier than host rock (*δ*^13^C_HR_) by 0.5–1‰ (Fig. 1c and Supplementary Table 3).

## Discussion

*δ*^34^S_HR_ of the Wanlong carbonates is within the range of sulphur isotopic compositions of Neoproterozoic CAS^13^. The greater values of *δ*^34^S_MT_ indicate that MT microspar was precipitated in the sulphate reduction zone in the sediment column, where ^32^S is preferentially removed from the porewater sulphate pool by sulphate reduction microbes^14^ (Supplementary Note 2). MT microspar precipitation in the MSR zone is also consistent with generally lower CAS concentrations in MT microspar than in host rock (Supplementary Fig. 6 and Supplementary Table 4).

*δ*^26^Mg_MT_ is related to the Mg isotopic composition of porewater (*δ*^26^Mg_pw_), from which MT microspar precipitates, and the relationship can be expressed as follows:


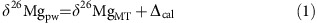


where Δ_cal_ is the fractionation associated with inorganic precipitation of low-Mg calcite and can be set at 2.2–2.7‰ (refs 15, 16). Thus, *δ*^26^Mg_pw_ is estimated to be between –0.6 and –1.1‰, within the range of seawater compositions in the past 70 million years^17^,^18^. Greater *δ*^26^Mg_HR_ values might be attributed to the partial dolomitization of host rock, because dolostone is systematically heavier than limestone in Mg isotopes^19^,^20^. On the other hand, as dolomite and other authigenic Ca carbonate formed in the sediment column would preferentially scavenge ^24^Mg from porewater^21^,^22^ (Supplementary Note 3), *δ*^26^Mg_pw_ would increase as dolomitization proceeds. It is estimated that 10–25 wt% of carbonate in the host rock of the Wanlong Formation is dolomitized (Fig. 1b), meaning *δ*^26^Mg_pw_ would increase by ∼2‰ (Supplementary Fig. 7). Had MT microspar in the Wanlong Formation precipitated after host rock dolomitization, seawater Mg isotopic composition would have to be between –2.6 and –3.1‰, which is even lower than the influx from carbonate weathering (–2.25‰; that is, the lower bound of riverine input)^18^. Thus, MT microspar precipitation must predate host rock dolomitization. This inference is also consistent with the petrographic observation that MT structures are often ptygmatically folded and sometimes brittly fractured^1^, suggesting that MT microspar was precipitated before host rock cementation. In this light, it is possible that the exclusive occurrence of MT structures in argillaceous carbonates^1^ may be related to clay minerals, which tend to delay host rock cementation^23^.

Thus, sedimentary evidence, S isotopes and Mg isotopes indicate that MT microspar precipitation must occur in unconsolidated sediments, within the MSR zone and before dolomitization. To generate MT structures, cracks must develop in unlithified sediments and gas expansion is a plausible mechanism to generate such cracks^2^,^4^. Here we explore the nature of the gases and the unique Proterozoic environments conducive for gas bubble formation in sediments.

During the early to middle Proterozoic, atmospheric oxygen level was extremely low (<1% present atmospheric level) and the deep ocean remained anoxic and sulphidic in places^24^,^25^,^26^,^27^. Sulphidic conditions were particularly common in Proterozoic continental margins^25^,^26^,^28^ and perhaps in epicratonal environments as well^29^,^30^. Although euxinia may have extended over <10% of global seafloor in mid-Proterozoic according to some estimates^27^, sulphidic waters might have had profound impacts on the Proterozoic Earth system. We propose that methyl sulphides might have been produced in significant quantities in sulphidic marine environments. Methyl sulphides are a group of volatile organic sulphur compounds, including dimethyl sulphide (CH_3_SCH_3_) and methanethiol (CH_3_SH), which are produced in modern marine and freshwater environments. Methyl sulphides can be produced either by the degradation of dimethylsulphoniopropionate in the surface ocean^31^ or by anaerobic methylation of hydrogen sulphide in sulphidic sediments^32^. Therefore, it is expected that the production of methyl sulphides would be enhanced in Proterozoic sulphidic marine environments, both in the water column and within sediments.

**Figure 2:**
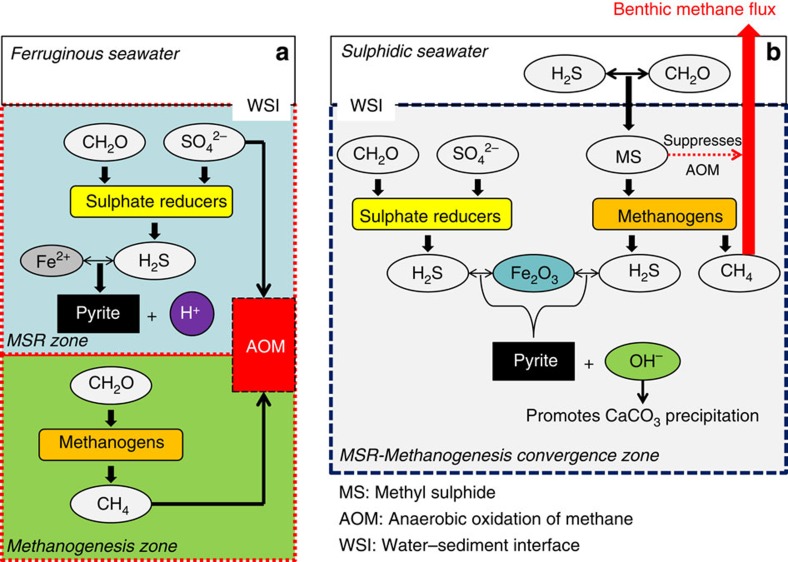
Schematic diagram showing geochemical reactions within marine sediments bathed beneath ferruginous and sulphidic seawaters. (**a**) Under ferruginous conditions, the MSR and methanogenesis zones are separated. Methanogens are outcompeted by sulphate-reducing bacteria, if both use competitive substrates (CH_2_O)_*n*_. Within the MSR zone, reaction between H_2_S and Fe^2+^ precipitates pyrite and generates H^+^, which lowers porewater pH. Most CH_4_ produced within methanogenesis zone is oxidized by sulphate at the base of MSR zone where AOM occurs. Thus, there is little benthic CH_4_ flux from marine sediments. (**b**) Under sulphidic conditions, methyl sulphides are produced within both water column and sediments. In sediments, methyl sulphides serve as a non-competitive substrate for methanogens, allowing MSR and methanogenesis to take place concurrently in the MSR-methanogenesis convergence zone. H_2_S and Fe_2_O_3_ react to produce pyrite and generate OH^–^, thus favouring CaCO_3_ precipitation. AOM is prohibited by methanethiol, allowing CH_4_ accumulation in sediments and significant benthic CH_4_ fluxes into atmosphere.

As volatile gases, methyl sulphides produced in water column tend to readily emit to atmosphere, but those generated within the sediments can serve as a non-competitive substrate for methanogens^33^,^34^,^35^. As sulphur-reducing microbes cannot use methyl sulphides but methanogens can, MSR and methanogenesis can co-occur simultaneously within sediments where methyl sulphides are present^36^, resulting in the convergence of the MSR and methanogenesis zones. In addition, anaerobic oxidation of methane (AOM) is inhibited by methyl sulphides such as methanethiol. With methane oxidation inhibited, CH_4_ can accumulate in sediments in significant quantity^37^, in sharp contrast to modern marine sediments, where the MSR zone lies invariably above the methanogenesis zone^38^, with intensive AOM at the base of MSR zone consuming most CH_4_ and consequently modern marine CH_4_ discharge accounting for only 2% of the global flux^39^ (Fig. 2).

We propose that the accumulation of the insoluble gas CH_4_ in the convergence zone provided a physical mechanism to generate cracks in unconsolidated sediments^2^,^4^. Furthermore, the geochemistry within the convergence zone where MSR and methanogenesis overlap could have facilitated the precipitation of calcite to fill such cracks. With the generally low concentrations of Fe^2+^ in sulphidic porewaters, pyrite formation would involve the reaction between H_2_S and Fe_2_O_3_. In fact, the host rock of MTCs in the Wanlong Formation contains an average of 0.42 wt% of pyrites (Supplementary Table 5). The overall reactions for pyrite formation fueled by MSR and methanogenesis using methanethiol and dimethyl sulphide can be described as follows:


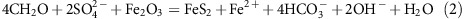



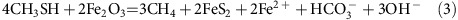



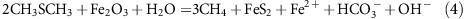


These reactions generate OH^−^ and 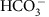, which elevate pH, increase porewater alkalinity and favour CaCO_3_ precipitation^40^,^41^,^42^. In addition, the dearth of calcite inhibitors such as Fe^2+^ and SO_4_^2–^ in sulphidic sediments would also promote rapid precipitation of CaCO_3_ (ref. 11).

**Figure 3:**
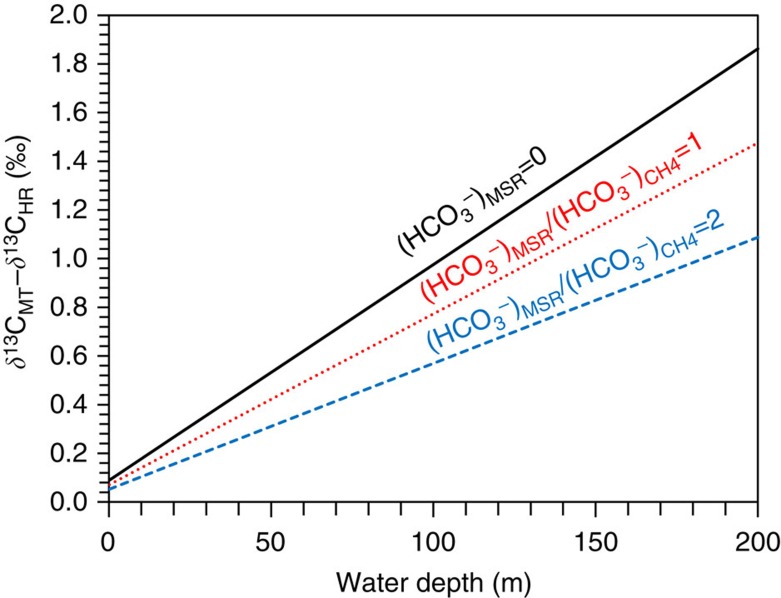
Geochemical model showing the carbon isotopic difference between MT microspar and host rock formed at different water depths. First, the amount of CH_4_ required to produce a unit volume of cracks at ambient pressure and temperature was estimated. The co-production of 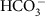 related to CH_4_ generation was then estimated and assumed to have been used fully for MT microspar precipitation. The methanogenic 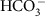 was inadequate to precipitate enough MT microspar to fill a unit volume and the shortage was made up by (1) pore water 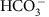 (black solid line); (2) 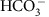 from MSR (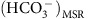) and methanogenesis (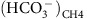) with a molar ratio of 1:1, and the remaining shortage fulfilled by pore water 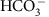 (red dotted line); or (3) 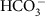 from MSR and methanogenesis with a molar ratio of 2:1, and the remaining shortage fulfilled by pore water 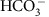 (blue dashed line). Porewater 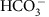 was assumed to have a *δ*^13^C value similar to that of host rock (that is, 1‰). The *δ*^13^C value of methanogenic 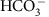 was estimated at +150‰, given a *δ*^13^C value of methyl sulphides at –30‰, a fractionation between CH_4_ and methyl sulphides at –60‰ and the production of 3/4 mole of CH_4_ and 1/4 mole of 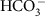 from each mole of methyl sulphides (equations (2) and (3)). The *δ*^13^C value of MSR 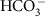 was assumed to be –30‰. The *x* axis represents water depths and the *y* axis indicates the isotopic difference between MT microspar and host rock (*δ*^13^C_MT_-*δ*^13^C_HR_).

*δ*^13^C_MT_ of the Wanlong carbonate is only slightly greater than *δ*^13^C_HR_ by 0.5–1‰ (Fig. 1c), similar to previous studies showing that MT microspar and host carbonate rock have nearly indistinguishable *δ*^13^C values^5^,^6^,^43^. To assess the extent of carbon isotope variation between MT microspar and host rock, we consider a simple model where methanogenesis produces sufficient CH_4_ to produce cracks that are immediately filled with MT microspar. To generate cracks by CH_4_ accumulation, gas pressure must be balanced with the hydrostatic pressure, which is dependent on water depth. Our calculation shows that methanogenesis alone does not generate sufficient bicarbonate (and MT microspar) to fill the cracks that would be created at reasonable water depths by the amount of CH_4_ it produces. Thus, MT microspar precipitation was probably supplemented by porewater bicarbonate (which would be isotopically similar to seawater bicarbonate and to *δ*^13^C_host_) and bicarbonate derived from sulphate reduction (Supplementary Note 4). To simplify our calculation, we consider the simplest situation in which bicarbonate derived from methanogenesis was entirely used in MT microspar precipitation, with additionally needed alkalinity coming from porewater (that is, a binary mixing model). Assuming that *δ*^13^C of methyl sulphides and carbon isotope fractionation during methanogenesis are –30‰ and –60‰ (ref. 44), respectively, mass balance consideration requires that *δ*^13^C of 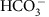 derived from methanogenesis be +150‰ based on equations (3) and (4). Our calculation shows that MT microspar precipitation at 100 m water depth would be ∼1‰ heavier than host rock (black solid line in Fig. 3) and methanogenesis-derived 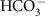 only accounts for <1% of MT microspar precipitation. Smaller isotopic difference between MT microspars and host rock would be expected if MSR-derived 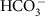 is involved (dashed lines in Fig. 3).

The disappearance of MTCs is coincident with the elevation of atmospheric oxygen levels at ∼750 Ma (refs 1, 45), suggesting a possible causal relationship. A direct consequence of ocean oxygenation and ventilation is the reduction of the areal coverage of euxinic waters and decrease in methyl sulphide production, which in turn would result in the spatial separation of the MSR and methanogenesis zones in sediments. As such, most CH_4_ was consumed at the base of MSR zone by AOM, preventing crack formation by CH_4_ accumulation. Furthermore, pyrite formation in ferruginous sediments through reaction with Fe^2+^ would generate protons, lowering porewater pH and favouring CaCO_3_ dissolution^46^,^47^. All these secular changes associated with atmospheric and oceanic oxygenation may have contributed to the disappearance of MT structure around 750 Ma.

CH_4_ accumulation in sediments also implies benthic CH_4_ discharge from marine sediments. The environmental impacts of benthic CH_4_ fluxes on the Proterozoic Earth system could potentially be profound. First, enhanced CH_4_ discharge would contribute to the persistently low atmospheric O_2_ levels in Proterozoic^25^,^48^. Second, strong benthic CH_4_ fluxes from continental margins would have contributed to the maintenance of an ice-free Earth in the middle Proterozoic. Finally, a significant reduction of CH_4_ discharge associated with the 750 Ma oxygenation event might have triggered the Neoproterozoic global glaciations. Thus, the Neoproterozoic oxygenation event may have had an impact on the secular distribution of sedimentary structures such as MT structure and the global climate system, as well as the rise of animals^45^.

## Methods

### Mg isotope analysis

Rock samples were split into two parts using a rock saw. A highly polished slab was prepared from one split, while a mirrored thin section was made from the counterpart. Sample powers were drilled from the polished slab using a hand-held micro-drill. The sampling procedure was guided by the petrographic observation of the corresponding thin sections. For Mg isotopic analysis, about 10–30 mg of powder was dissolved in 0.5 N acetic acid in a 15-ml centrifuge tube. Tubes were placed in an ultrasonic bath for 30 min, to allow complete dissolution of carbonate components. After centrifuging, supernatant was collected for column chemistry and elemental composition analysis.

Mg was purified using cation-exchange chromatography. The detailed procedure of column chemistry was reported in Shen, *et al.*^49^ and Huang *et al.*^20^ Mg was purified in two steps. Column 1 was designated to separate Mg from Ca. Mg was eluted by 4 ml of 10 N HCl, whereas Ca was retained in resin. Column 2 was used to separate Mg from all other elements. Na, Al, Fe and K were sequentially eluted using 1 N HCl, 1 N HNO_3_+0.5 N HF and 1 N HNO_3_, whereas Mg was collected using 5 ml of 2 N HNO_3_. To obtain a pure fraction of Mg, sample solutions passed through column 1 twice, followed by three passes through column 2. After column chemistry, Ca/Mg, Na/Mg, Al/Mg and Fe/Mg ratios were <0.05 and the Mg recovery rate was better than 99%.

Mg isotopic ratios were measured on a Thermo Scientific Neptune Plus high-resolution multicollector inductively coupled plasma mass spectrometry at the Isotope Laboratory in China University of Geosciences, Beijing. The standard-sample bracketing method was used to correct the instrumental mass bias and drift. An in-house solution (FZT) was used as the working standard. Analyses were performed in the low-resolution mode, simultaneously measuring ^26^Mg, ^25^Mg and ^24^Mg isotopes. Mg isotope ratios are reported by the delta notation as ‰ deviation relative to the DSM3 standard^50^:


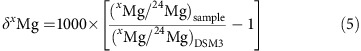


where *x* refers to 25 or 26.

The internal precision was determined on the basis of ≥3 repeated runs of the same sample solution during a single analytical session and is better than ±0.10‰ (2 s.d.). The accuracy is determined by the measurements of synthetic solution (GSB-Mg) and USGS basalt standards (BCR-2). Multiple analyses of the synthetic solution (GSB-Mg) yield *δ*^26^Mg values ranging from –2.07 to –2.04‰, which is consistent with the preferred value of –2.05±0.05‰ (2*σ*). *δ*^26^Mg of BCR-2 is –0.17±0.06‰ (2*σ*), consistent with the published values^51^,^52^,^53^,^54^.

### Sulphur isotope analysis

Traditional CAS extraction procedure typically requires >20 g of carbonate powder. Thus, it is impossible to collect enough sample powder from MT microspar without contamination from the host rock using the traditional method, because MT cracks are typically a few millimetres in width. To analyse CAS of MT microspar, we devised a new extraction procedure that only requires ∼1 g of carbonate powders for each sample. The validity of the new procedure was verified by analysing the same carbonate sample by using both the traditional and new procedures. Powders were carefully drilled from MT microspar only to a shallow depth so as to avoid the potential contamination from host rock. Often, multiple MT cracks in a polished slab were drilled to collect enough powder for CAS extraction. Sample powder was placed in a 50-ml centrifuge tube and were treated with 10% NaCl solution for 24 h to dissolve non-CAS sulphate. After supernatant removal, residues were washed with deionized water for three times. The above cleaning procedures were repeated at least three times, to ensure complete removal of non-CAS sulphate. The cleaned sample powder was dissolved in 40 ml of 3 N HCl. After 1 h of reaction, reaction tubes were centrifuged and the supernatants were collected. About 1–2 mg of nano-SiO_2_ was added into the centrifuge tube and then 10 ml of saturated BaCl_2_ was added to precipitate sulphate as barite. The use of nano-SiO_2_ was to facilitate barite collection. Barite precipitation was allowed to proceed for 48 h. After centrifuging, barite precipitate was washed by DI water for three times, to remove residual HCl, and then dried in an oven.

Sulphur isotopic compositions were measured at Indiana University on a Finnigan Delta V advantage gas source mass spectrometry fitted with a peripheral Costech elemental analyser for on-line sample combustion. Sulphur isotope compositions are reported as ‰ deviation from V-CDT, *δ*^34^S=(*R*_sample_/*R*_V-CDT_−1) × 1,000, where *R* is the ratio of ^34^S/^32^S. Analytical error is ±0.1‰ (1*σ*) as determined from repeated analyses of samples and laboratory standards. The analytical results were calibrated using the standard NBS-127 (20.3‰) and three internal standards: a silver sulphide (ERE-Ag_2_S: −4.3‰), a chalcopyrite (EMR-CP: +0.9‰) and a barite (PQB2: +40.5‰).

### Inductively coupled plasma optical emission spectrometer analysis

Elemental compositions were determined at Peking University on a Spectro Blue Sop inductively coupled plasma optical emission spectrometer fitted with a Water Cross-flow nebulizer. All analyses were calibrated by a series of gravimetric standards with different concentrations (ranging from 0.1 to 10 p.p.m.) that were run before sample measurements and between every 20 samples. The external reproducibility for the major and minor elements (Na, Mg, Al, K, Ca, Fe, Mn, Sr and S) is ±2%.

## Additional information

**How to cite this article**: Shen, B. *et al.* Molar tooth carbonates and benthic methane fluxes in Proterozoic oceans. *Nat. Commun.* 7:10317 doi: 10.1038/ncomms10317 (2016).

## Supplementary Material

Supplementary Information Supplementary Figures 1-7, Supplementary Tables 1-5, Supplementary Notes 1-4 and Supplementary References
