# Supplementary material for: Molar tooth carbonates and benthic methane fluxes in Proterozoic oceans
Source: Nat Commun. 2016 Jan 7;7:10317. doi: 10.1038/ncomms10317 (PMC4729840; doi:10.1038/ncomms10317)
Supplement: Supplementary Information — Supplementary Figures 1-7, Supplementary Tables 1-5, Supplementary Notes 1-4 and Supplementary References [file ncomms10317-s1.pdf]

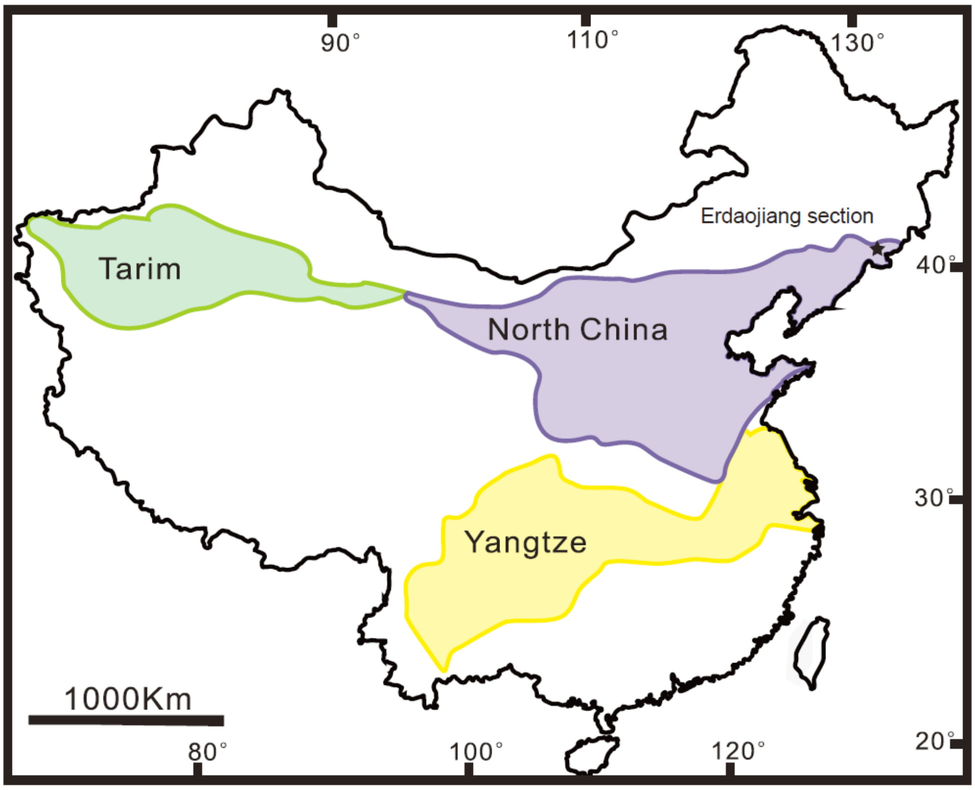


**Supplementary Figure 1:** Simplified map showing the sampling locality (the Erdaojiang section, blue star) in the northeastern margin of North China Block.

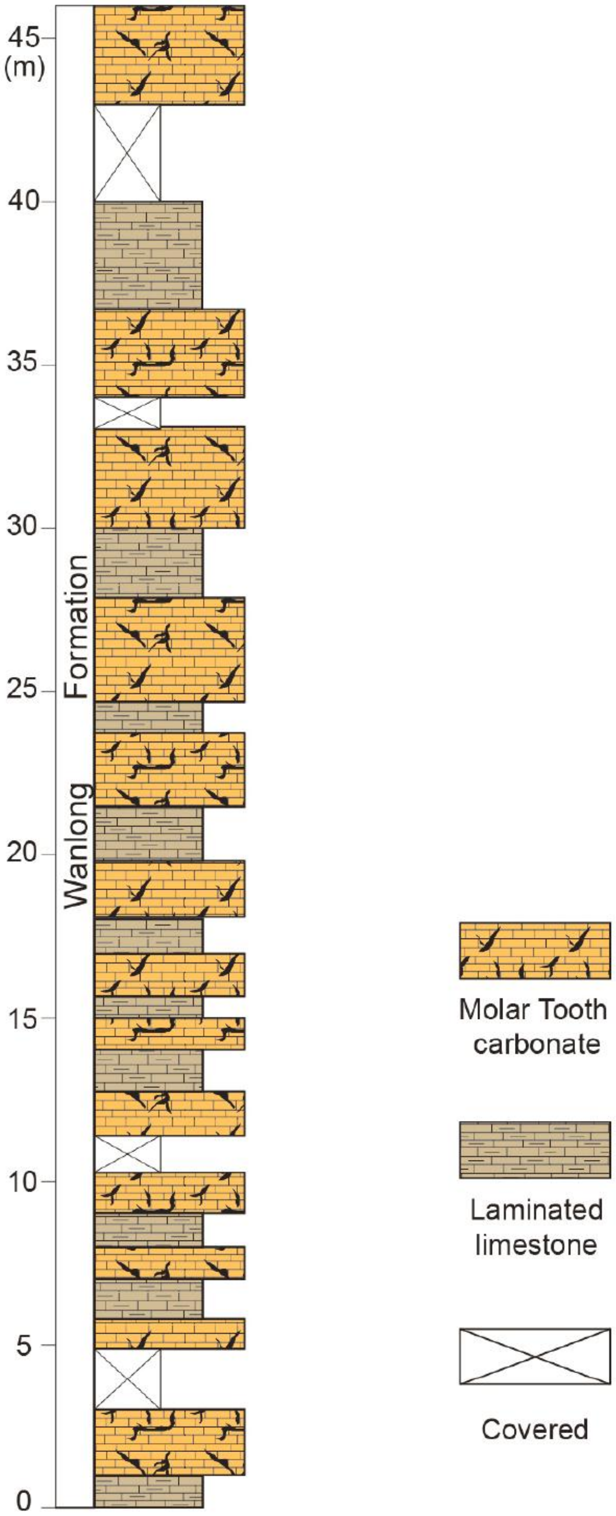


**Supplementary Figure 2:** Stratigraphic column of the lower member of the Wanlong Formation at the Erdaojiang section, southern Jilin Province, North China.

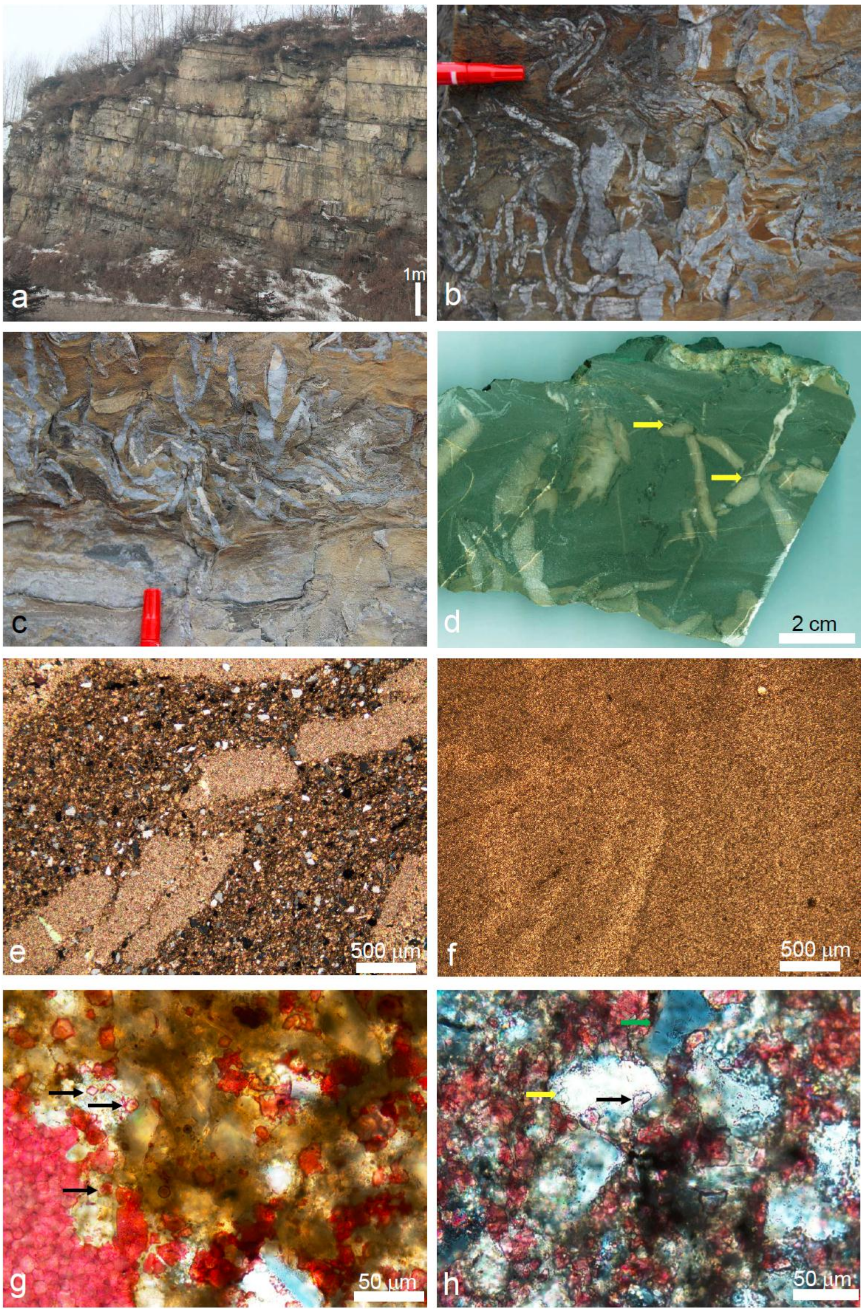


**Supplementary Figure 3: Field photographs and transmitted light photomicrographs.** a: Field photograph showing the Wanlong Formation at the Erdaojiang section, southern Jilin Province, North China. b and c: Outcrop photos showing MT (grey in color) in argillaceous limestone (brown in color). d: Polished hand specimen showing the sharp boundary between MT microspar and host rock, and cross-cutting relationship among MT structures (yellow arrows). e: Photomicrograph showing MT calcispar and argillaceous host rock under transmitted light. f: Photomicrograph showing MT microspar. g: Photomicrograph showing contact between MT microspar (lower left, stained red using alizarin red) and host rock. Note residual microspar and inclusions (black arrows) in dolomite crystals. h: Photomicrograph of argillaceous host rock. Note abundant detrital quartz (green arrow) and rhombic dolomite crystals (yellow arrow). Abundant inclusions (black arrow) are also present in dolomite crystals. In (g and h), calcite is stained red using alizarin red, and dolomite is not stained.

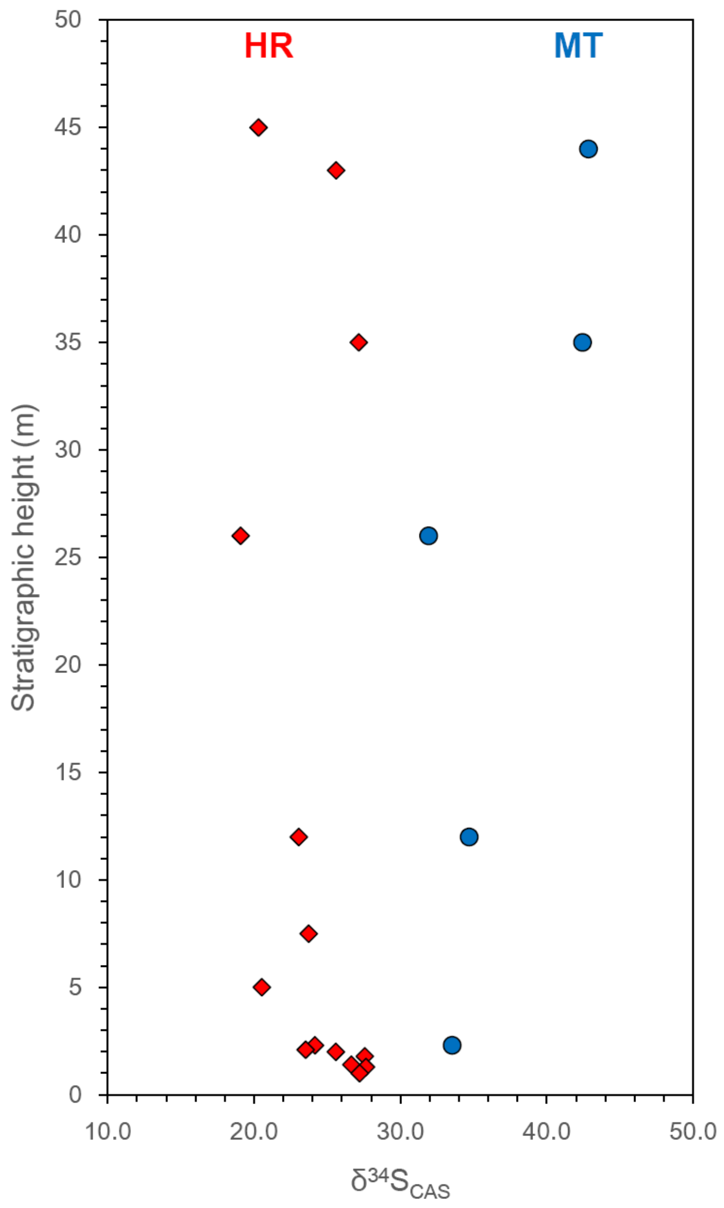


**Supplementary Figure 4:** Stratigraphic profile of sulfur isotopic compositions of carbonate associated sulfate extracted from MT microspar (blue circles) and host rock (red diamonds).

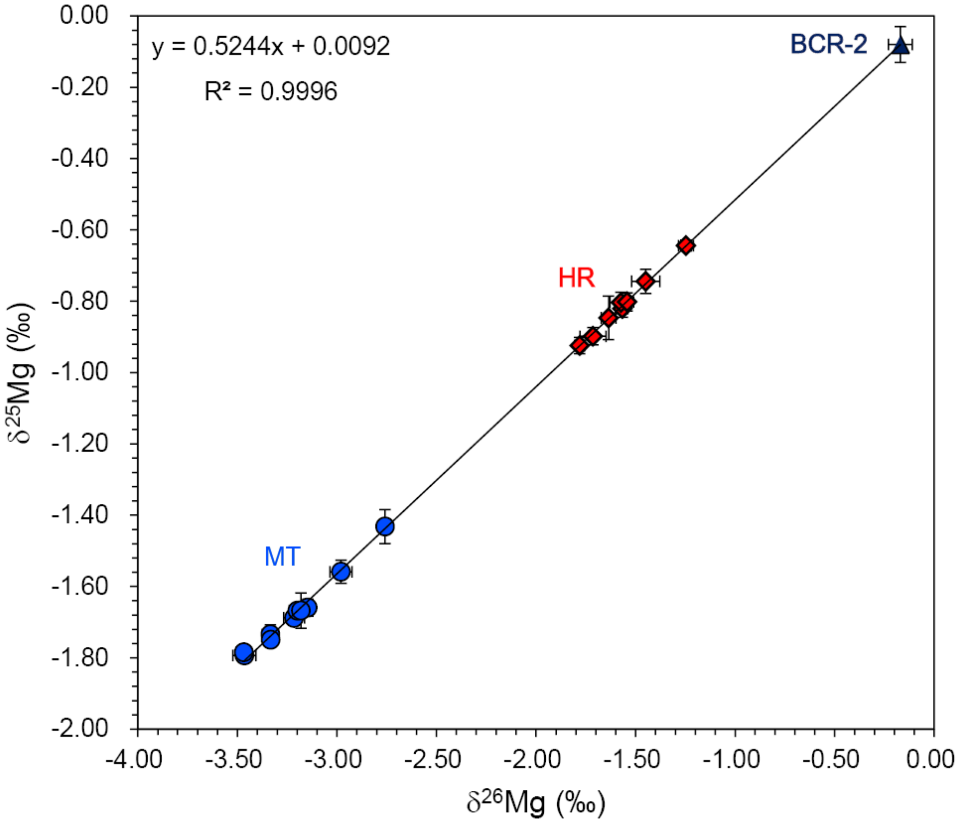


**Supplementary Figure 5:** $\delta^{26}$Mg–$\delta^{25}$Mg cross-plot of MTC samples and standard (BCR-2). Solid line shows the regression line of all data.

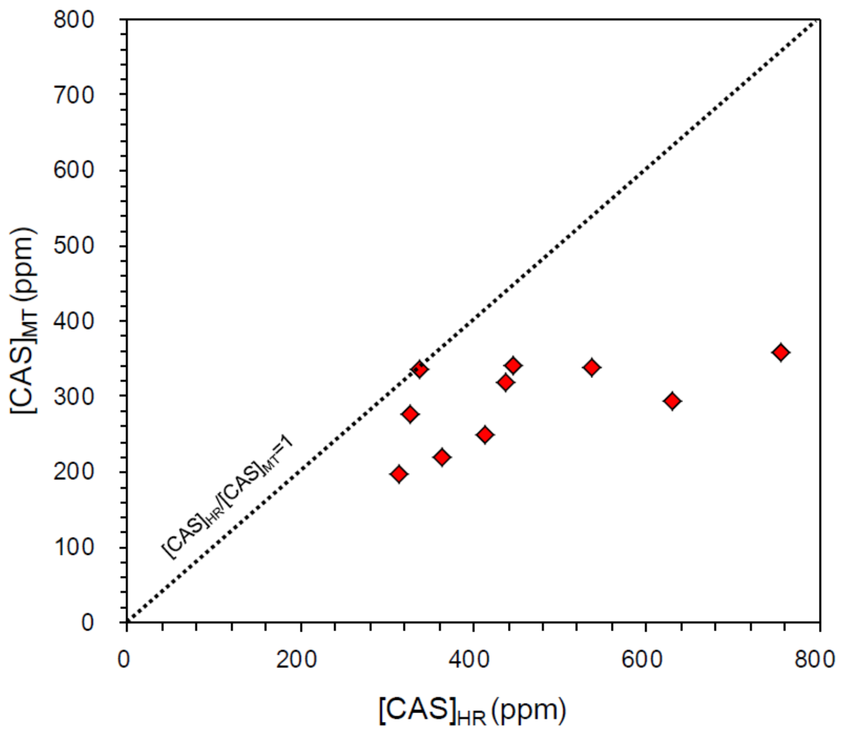


**Supplementary Figure 6: CAS concentration.** Crossplot showing carbonate associated sulfate (CAS) content of host rock (x-axis) vs. MT microspar (y-axis). The dotted line represents $[CAS]_{HR}/[CAS]_{MT} = 1$. All data are plotted below the dotted line, suggesting that CAS concentration in MT microspar is systematically lower than that in host rock.

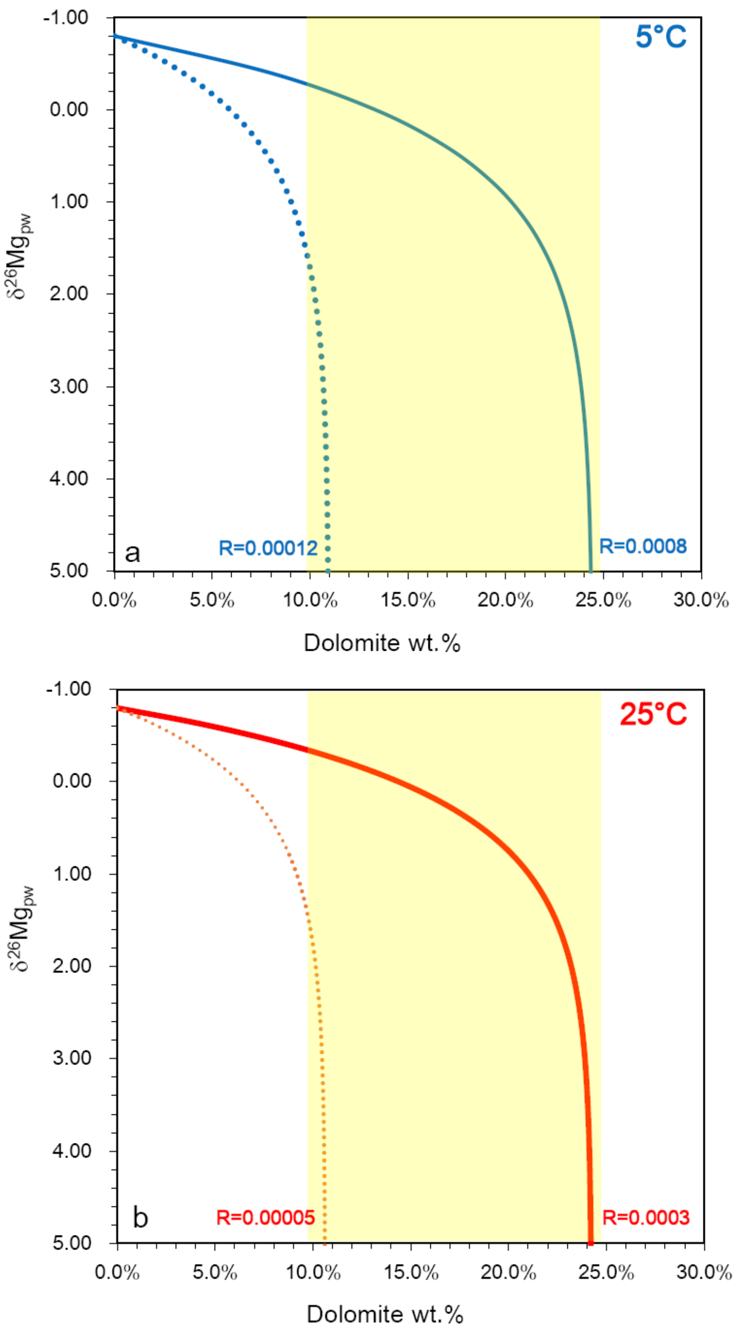


**Supplementary Figure 7: Mg isotope modeling result.** Relationship between dolomite content and porewater Mg isotopic composition ($\delta^{26}Mg_{pw}$), showing that dolomitization drives $\delta^{26}Mg_{pw}$ to greater values. The dashed and solid lines represent the lower (10%) and upper (25%) bounds of dolomite content, which were calculated by adjusting rate constant (R). Panels (a) and (b) represent the dolomitization temperature of $5^{o}$ C and $25^{o}$ C, respectively. Temperature would affect the diffusion coefficient ($D_{Mg}$) and isotopic fractionation during dolomite formation ($\Delta_{dol}$). $D_{Mg}$ at 5 ℃ and 25 ℃ is set at $1.8\times10^{-10}$ and $3.3\times10^{-10}$ $m^2s^{-1}$, respectively. $\Delta_{dol}$ at 5 ℃ and 25 ℃ is set at 2.0‰ and 1.8‰ (Ref. [18]), respectively. The calculation assumes $\delta^{26}Mg_0 = \delta^{26}Mg_{sw} = -0.8$‰ of modern seawater value, seawater Mg concentration of 100 mM[24], and sedimentation rate of 5 cm/ky[20].

**Supplementary Table 1:** Mg isotopic compositions of MT microspar and host rock samples from the Wanlong Formation, southern Jilin Province, North China. MT: MT microspar; 2SD: 2 standard deviations of the mean.

| Sample ID | Description | $\delta^{25}$Mg (‰) | 2SD | $\delta^{26}$Mg (‰) | 2SD | Mg/Ca (molar) | n |
|---|---|---|---|---|---|---|---|
| | | | | | | | |
| MT1-1 | Host rock | -0.64 | 0.02 | -1.25 | 0.04 | 0.104 | 3 |
| MT1-3 | MTC | -1.79 | 0.01 | -3.46 | 0.06 | 0.010 | 3 |
| MT1-4 | MTC | -1.78 | 0.02 | -3.47 | 0.04 | 0.009 | 3 |
| MT1-5 | MTC | -1.69 | 0.01 | -3.22 | 0.05 | 0.013 | 3 |
| MT1-7 | Host rock | -0.92 | 0.02 | -1.78 | 0.02 | 0.217 | 3 |
| MT1-8 | Host rock | -0.80 | 0.03 | -1.57 | 0.06 | 0.112 | 3 |
| MT1-10 | MTC | -1.73 | 0.03 | -3.33 | 0.04 | 0.010 | 3 |
| MT1-11 | Host rock | -0.90 | 0.02 | -1.71 | 0.07 | 0.270 | 3 |
| MT2-1 | Host rock | -0.74 | 0.03 | -1.45 | 0.07 | 0.115 | 3 |
| MT2-2 | MTC | -1.67 | 0.02 | -3.20 | 0.02 | 0.012 | 3 |
| MT2-3 | Host rock | -0.80 | 0.02 | -1.54 | 0.03 | 0.153 | 3 |
| MT2-5 | MTC | -1.66 | 0.02 | -3.15 | 0.02 | 0.014 | 3 |
| MT2-5(dup) | MTC | -1.67 | 0.05 | -3.18 | 0.06 | - | 3 |
| MT2-7 | MTC | -1.56 | 0.03 | -2.98 | 0.06 | 0.031 | 3 |
| MT2-8 | Host rock | -0.85 | 0.06 | -1.64 | 0.04 | 0.096 | 3 |
| MT2-9 | MTC | -1.75 | 0.02 | -3.33 | 0.01 | 0.011 | 3 |
| MT2-10 | MTC | -0.82 | 0.02 | -1.57 | 0.03 | 0.169 | 3 |
| MT2-11 | MTC | -1.43 | 0.05 | -2.76 | 0.02 | 0.011 | 3 |
| BCR-2 | Standard | -0.08 | 0.05 | -0.17 | 0.06 | - | 3 |

**Supplementary Table 2:** S isotopic compositions of carbonate associated sulphate extracted from MT microspar and host rock samples of the Wanlong Formation, southern Jilin Province, North China. MT: MT microspar.

| Sample ID | Description | $\delta^{34}S_{CAS}$ (‰) |
|---|---|---|
| | | |
| MT-10 | MT | 34.7 |
| MT-8 | MT | 42.4 |
| MT-11 | MT | 31.9 |
| 15EDJ-8MT | MT | 33.5 |
| 15EDJ-18MT | MT | 42.8 |
| MT-5 | Host rock | 19.1 |
| MT-9 | Host rock | 23.1 |
| 15EDJ-1 | Host rock | 27.2 |
| 15EDJ-3-1 | Host rock | 27.6 |
| 15EDJ-3-2 | Host rock | 26.6 |
| 15EDJ-5 | Host rock | 27.5 |
| 15EDJ-6 | Host rock | 25.6 |
| 15EDJ-7 | Host rock | 23.5 |
| 15EDJ-8 | Host rock | 24.1 |
| 15EDJ-11 | Host rock | 20.5 |
| 15EDJ-12 | Host rock | 23.7 |
| 15EDJ-17 | Host rock | 27.2 |
| 15EDJ-19 | Host rock | 25.6 |
| 15EDJ-20 | Host rock | 20.3 |

**Supplementary Table 3:** C and O isotopic compositions of MT microspar and host rock samples of the Wanlong Formation, southern Jilin Province, North China. MT: MT microspar.

| Sample ID | Description | $\delta^{13}C$ (‰) | $\delta^{18}O$ (‰) |
|---|---|---|---|
| A1 | MTC | 1.63 | -5.06 |
| A2 | MTC | 1.41 | -6.15 |
| A3 | Host rock | 1.05 | -7.52 |
| A4 | Host rock | 1.10 | -7.44 |
| A5 | Host rock | 1.08 | -7.38 |
| B1 | MTC | 1.86 | -7.60 |
| B2 | MTC | 1.97 | -7.06 |
| B3 | Host rock | 0.99 | -7.05 |
| B4 | Host rock | 1.02 | -7.33 |
| C1 | MTC | 1.33 | -7.69 |
| C2 | MTC | 1.44 | -7.36 |
| C3 | Host rock | 1.08 | -7.46 |
| C4 | Host rock | 0.99 | -7.61 |
| D1 | MTC | 1.94 | -7.82 |
| D2 | Host rock | 0.95 | -7.20 |
| D3 | Host rock | 1.03 | -7.23 |

**Supplementary Table 4:** CAS concentrations of MT microspar and host rock samples of the Wanlong Formation, southern Jilin Province, North China. MT: MT calcispar.

| Sample ID | $[CAS]_{MT}$ (ppm) | $[CAS]_{HR}$ (ppm) |
|---|---|---|
| | | |
| EDJ-3-1 | 358 | 757 |
| EDJ-3-2 | 342 | 448 |
| EDJ-5 | 294 | 632 |
| EDJ-7 | 339 | 538 |
| EDJ-8 | 317 | 439 |
| EDJ-9 | 196 | 315 |
| EDJ-11 | 276 | 328 |
| EDJ-13 | 248 | 414 |
| EDJ-17 | 337 | 339 |
| EDJ-20 | 218 | 364 |

**Supplementary Table 5:** Siliciclastic contents and pyrite concentrations in host rock samples of the Wanlong Formation, southern Jilin Province, North China.

| Sample No. | Siliciclastic content (wt.%) | Pyrite in siliciclasts (wt.%) | Pyrite in bulk sample (wt.%) |
|---|---|---|---|
| 15EDJ-1 | 42.4 | 1.14 | 0.48 |
| 15EDJ-2 | 8.3 | 2.18 | 0.18 |
| 15EDJ-3-1 | 51.3 | 2.66 | 1.37 |
| 15EDJ-3-2 | 30.3 | 1.32 | 0.40 |
| 15EDJ-4 | 46.8 | 0.85 | 0.40 |
| 15EDJ-5 | 55.3 | 0.98 | 0.54 |
| 15EDJ-6 | 43.2 | 1.41 | 0.61 |
| 15EDJ-7 | 10.1 | 0.49 | 0.05 |
| 15EDJ-8 | 30.6 | 1.13 | 0.35 |
| 15EDJ-9 | 18.6 | 1.75 | 0.33 |
| 15EDJ-11 | 18.2 | 1.55 | 0.28 |
| 15EDJ-12 | 49.2 | 1.23 | 0.61 |
| 15EDJ-13 | 55.4 | 0.08 | 0.05 |
| 15EDJ-14 | 24.3 | 2.42 | 0.59 |
| 15EDJ-17 | 19.5 | 1.12 | 0.22 |
| 15EDJ-19 | 34.2 | 1.06 | 0.36 |
| 15EDJ-20 | 25.1 | 1.08 | 0.27 |
| Average | 33.1 | 1.32 | 0.42 |

# Supplementary note 1: Geological background

Molar Tooth carbonate (MTC) samples were collected from the Neoproterozoic Wanlong Formation at the Erdaojiang section in southern Jilin Province, North China (Supplementary Fig. 1). The Erdaojiang section is located in the northeastern (in the present orientation) margin of the North China Block (Supplementary Fig. 1). The Neoproterozoic succession in this region consists of, in ascending order, the Qiaotou, Wanlong, Badaojiang, and Qinggouzi formations. The Wanlong Formation conformably overlies greyish green to yellowish green shale of the Qiaotou Formation, and is unconformably underlain by the stramatolitic limestone of the Badaojiang Formation[1]. The Wanlong Formation is subdivided into three lithological members. The lower member is composed of light grey to yellowish grey, medium- to thick-bedded lime mudstone intercalated with fine-laminated limestone (Supplementary Fig. 2). The middle member is dominated by the alternating of dark grey nodular limestone and laminated limestone. The upper member consists of yellowish green calcareous shale and argillaceous limestone. Abundant MTC occurs in the thick-bedded argillaceous lime mudstone of the lower member Supplementary Fig. 2, 3a).

In the Wanlong Formation, MTC abundantly occurs in the thick-bedded, argillaceous lime mudstone of the lower member. In outcrops, the argillaceous host rock is weathered to yellow color, while the MT microspar that fills the MT cracks is light grey in color (Supplementary Fig. 3b, c). MT cracks typically orient vertically or obliquely relative to the bedding plane, are often deformed ptygmatically and sometimes fractured brittly, and can cross-cut with each other (Supplementary Fig. 3b-d). The width of the MT cracks ranges from >1 cm to <1 mm. In outcrops and hand specimens, MT microspars have sharp contacts with host rock (Supplementary Fig. 3b-d). Under a microscope, MT cracks are filled by equant microcrystalline calcite crystals (MT microspar) ranging from 10 to 20 μm in size (Supplementary Fig. 3e-g). The ptygmatic deformation and brittle fracture of MT structures suggest that MT structures were coherent or rigid upon sediment compaction and thus microspar precipitation must have predated cementation of host sediments.

The argillaceous host rock contains abundant siliciclastic materials (with an average of 33.1

wt.%, n=17, Supplementary Table 5), dominated by detrital quartz (green arrow in Supplementary Fig. 3h) and clay minerals. Host rock also contains substantial amount of pyrites (with an average of 0.42 wt.% in bulk sample, Supplementary Table 5). The calcareous component of the host rock includes micritic calcite and dolomite. The euhedral dolomite crystals within host rock are normally less than 100 μm in size, and the crystal margins are eroded due to dissolution (yellow arrow in Supplementary Fig. 3h). The abundant inclusions and engulfment of micrite within rhombic dolomite crystals (black arrows in Supplementary Fig. 3g, h) indicate that these are replacive dolomite crystals. Based on the measurement of Mg/Ca ratios, dolomite accounts for 10% to 25% of total calcareous content (Supplementary Table 1, Supplementary Fig. 1b).

## Supplementary note 2: Sulphur isotope systematics in sediments

Significant isotopic fractionations are associated with various redox reactions of sulphur species. One of the most important reactions within marine sediments is microbial sulphate reduction (MSR), in which organic matter is anaerobically oxidized by sulphate reducing microbes (SRM) to bicarbonate, while sulphate, as the electron receptor, is reduced to $H_2S$[2,3]. MSR takes place in the MSR zone in sediments, and preferentially utilizes $^{32}S$-enriched sulphate, leaving porewater sulphate enriched in $^{34}S$[3]. Furthermore, trace amount of sulphate is incorporated into carbonate lattice during carbonate precipitation. Such trace sulphate is called the carbonate associated sulphate or CAS[4]. For marine carbonate, sulphur isotopic compositions of CAS record the seawater value; thus, CAS have been widely used as a proxy for the isotopic compositions of seawater sulphate[5,6]. If carbonate was precipitated from sediment porewater (i.e., authigenic carbonate), its CAS sulphur isotopic composition records the porewater sulphate value. Because of preferential utilization of $^{32}S$ in MSR, CAS of authigenic carbonates precipitated in the MSR zone are expected to have larger $\delta^{34}S$ values than carbonate directly precipitated in seawater.

## Supplementary note 3: Magnesium isotope systematics in sediments

With the development of high resolution Mg isotope analysis technique, Mg isotopes become

an important approach in the study of carbonate[7-12]. There are significant fractionations in Mg isotopes during biogenic and abiotic carbonate precipitation[13-16]. Because authigenic Ca-carbonate precipitation preferentially utilizes $^{24}$Mg and leaves $^{26}$Mg in porewater, the porewater Mg isotopes become progressively heavier with increasing sediment depth[10,11]. Thus, Mg isotopes can be used to constrain the authigenic precipitation of carbonate minerals. To a first-order approximation, carbonate precipitated in seawater should be isotopically lighter than authigenic carbonate precipitated within calcareous sediments, and early authigenic carbonate formed at shallower depth of sediments would be enriched in $^{24}$Mg as compared with late authigenic carbonate precipitated in deeper sediments[10]. In contrast, a reversed trend is expected in siliciclastic sediments, where authigenic clay mineral formation predominates and $^{26}$Mg is preferentially utilized during clay formation, leaving lighter Mg in sediment porewater[10].

Dolomite formation normally takes place in marine sediments (dolomitization), and can be constrained by Mg isotopes[9,17]. Because dolomite precipitation preferentially utilize $^{24}$Mg, dolomitization would drive porewater enrichment in $^{26}$Mg[9,10,18]. We can use the diffusion-advection-reaction (DAR) model[17] to simulate porewater Mg isotopic compositions during dolomitization. The one-dimensional DAR model can be expressed by the following equation:

$$\frac{\partial[^{i}\mathrm{Mg}]}{\partial t} = D_{Mg}\frac{\partial^2[^{i}\mathrm{Mg}]}{\partial z^2} - s\frac{\partial[^{i}\mathrm{Mg}]}{\partial z} - R_i[^{i}\mathrm{Mg}] \qquad \text{(S-1)}$$

where $[^{i}\mathrm{Mg}]$ is the porewater concentration of $^{24}$Mg or $^{26}$Mg, z is the sediment depth below the water-sediment interface (WSI), s is the sedimentation rate, $D_z$ is the vertical diffusion coefficient, $R_i$ is the rate constant for $^{24}$Mg or $^{26}$Mg removal from porewater.

The steady state solution (i.e., $\frac{\partial[\mathrm{Mg}]}{\partial t} = 0$, and $D_{Mg}$, $R_i$ and s are constants) for Eq. S-1 can be written as:

$$[^{i}\mathrm{Mg}] = [^{i}\mathrm{Mg}_0]e^{\frac{[s-(s^2+4R_iD_{Mg})^{\frac{1}{2}}]}{2D_{Mg}}\times z} \qquad \text{(S-2)}$$

where $[^{i}\mathrm{Mg}_0]$ is the Mg concentration at WSI that equals to the seawater Mg concentration.

The isotopic fractionation during dolomite formation is related to the difference in the

reaction rates between $^{24}Mg$ and $^{26}Mg$, and follows the following relationship:

$$\frac{R_{Mg}^{26}}{R_{Mg}^{24}} = \alpha_{24}^{26} \quad \text{(S-3)}$$

$\alpha_{24}^{26}$ is related to the isotopic fractionation ($\Delta_{dol}$) by $\Delta_{dol} = 1000 \times (\alpha_{24}^{26} - 1)$. The porewater Mg isotopic composition ($\delta^{26}Mg_{pw}$) can be calculated from [$^{24}Mg$] and [$^{26}Mg$] by the following equation:

$$\delta^{26}Mg_{pw} = [Ln([^{26}Mg]/[^{24}Mg]) - Ln([^{26}Mg]_{DSM3}/[^{26}Mg]_{DSM3})] \times 1000 \quad \text{(S-4)}$$

In Eq. S-4, DSM3 denotes the standard for Mg isotopes [19]. At WSI, seawater Mg isotopic composition can be calculated as:

$$\delta^{26}Mg_0 = [Ln([^{26}Mg]_0/[^{26}Mg]_0]) - Ln([^{26}Mg]_{DSM3}/[^{26}Mg]_{DSM3}])] \times 1000 \quad \text{(S-5)}$$

The amount of Mg being transferred into dolomite ($M_{dol}$) can be calculated as follows:

$$M_{dol} = \int_0^{\infty} \frac{[Mg]R}{v} dz \quad \text{(S-6)}$$

Dolomite content can be calculated from $M_{dol}$ by assuming carbonate rocks with the density of 2.5 g/cm$^3$. Dolomite content is set to be between 10 wt.% and wt.25% according to measured Mg/Ca ratio (Fig. 1a), R is approximated by $R_{24}$ or the rate constant of $^{24}Mg$—the dominant Mg isotope, and R is tuned to satisfy 10 wt.% or 25 wt.% of dolomite.

In the DAR model, porewater Mg isotopic profiles are controlled by the following parameters: $D_{Mg}$, s, $R_{Mg}$, $[Mg]_0$, $\Delta_{dol}$, and $\delta^{26}Mg_0$. Diffusion coefficient for $Mg^{2+}$ in seawater ($D_{sw}$) is temperature dependent, and varies from $3.26 \times 10^{-10}$ to $6.55 \times 10^{-10}$ $m^2s^{-1}$ at temperatures between 5 ℃ and 25 ℃. Diffusion coefficient for $Mg^{2+}$ in sediments porewater ($D_{sed}$) is smaller than its seawater value, and can be calculated by: $D_{sed} = D_{sw}/(1 - In(\emptyset^2))$, in which $\emptyset$ is the porosity. $D_{sed}$ for $Mg^{2+}$ in sediments with 60% porosity is $1.8 \times 10^{-10}$ and $3.3 \times 10^{-10}$ $m^2s^{-1}$ at 5 ℃ and 25 ℃, respectively. The depositional rate (s) of normal marine non-reef carbonates is in the range of 1~10 cm/ky[20]. Here, we choose the intermediate value of 5 cm/ky.

$[Mg]_0$ in the Ediacaran seawater is estimated to be 100 mM[21]. The absolute value of the rate constant for dolomitization is unconstrained. Accordingly, we treat $R_{Mg}$ as a tunable parameter in

our model. Fractionation for dolomite formation is also temperature dependent. Based on Li, et al. [18], $\Delta_{dol}$ is 2.0‰ and 1.8‰ at 5°C and 25 ℃, respectively.

For the MTC of the Wanlong Formation, host rock contains 10 – 25 wt.% of dolomite. Our calculation shows that even 10 wt.% of dolomitization means that porewater Mg isotopic values would evolve to ~1.2‰, about 2‰ higher than the presumed seawater value of −0.8‰ (Supplementary Fig. 7).

## Supplementary note 4: Carbon isotope systematics of MT microspar

To generate cracks in sediments by gas expansion, the gas pressure must be equal to or greater than the hydrostatic pressure[22], which is a function of water depth and sediment loading. Assuming the crack formation at a very shallow depth just below the water-sediment interface, sediment loading is negligible and the hydrostatic pressure is a function of water depth h. For an ideal gas, gas pressure (P), volume (V), and temperature (T) follow the Ideal Gas Law:

$$PV = nRT \qquad \text{(S-7)}$$

where n is the amount of gas (in moles), and R is the ideal gas constant. At constant temperature (isothermal), pressure and volume are inversely correlated. The hydrostatic pressure can be calculated from the Pascal's Law, and is determined by the water depth (h) and density of seawater (ρ):

$$P = h \times \rho \times g \qquad \text{(S-8)}$$

where g is the acceleration of gravity. At standard condition (with the pressure of 1 atmosphere or $10^5$ pa and temperature of $25^o$ C), the volume of one mole of ideal gas (the molar volume) is 22.4 L. With increasing pressure, the molar volume of ideal gas decrease proportionally. For example, at 100 m water depth (with pressure of ~10 atm from the water loading plus 1 atm from atmosphere), the molar volume is ~ 2L.

Thus, the hydrostatic pressure at 100 m water depth would be equilibrium with 1 mole of $CH_4$ at 2L in volume. If the gas cracks were completely filled by calcite, precipitation of ~ 5 kg of calcite (with density of $2.5 g/cm^3$) or 50 mole of bicarbonate is required. On the other hand, if

methane is generated by methanogenesis using methanethiol and dimethyl sulfide as substrate, one mole of methane formation would associate with 0.33 mole of $HCO_3^-$ production (equation 3 and 4). Thus, methanogenesis-derived $HCO_3^-$ can only account for 1/150 of $HCO_3^-$ that is required for crack filling, and additional $HCO_3^-$ should be sourced from sediment porewater or MSR (equation 2).

In a simple mixing model, where MT microspar is derived from methanogenesis-derived and porewater $HCO_3^-$, the isotopic composition of crack-filling MT microspar can be expressed by the binary mixing model:

$$\delta^{13}C_{MT} = f_{CH4} \times \delta^{13}C_{HCO_3^-}^{CH4} + (1 - f_{CH4}) \times \delta^{13}C_{HCO_3^-}^{pw} \quad \text{(S-9)}$$

where $\delta^{13}C_{MT}$, $\delta^{13}C_{HCO_3^-}^{CH4}$, $\delta^{13}C_{HCO_3^-}^{pw}$ are the carbon isotopic compositions of MT microspar, methanogenesis-derived $HCO_3^-$, and porewater $HCO_3^-$. $f_{CH4}$ is the fraction of methanogenesis-derived bicarbonate. At 100 m water depth, $f_{CH4}$=1/150.

$\delta^{13}C_{HCO_3^-}^{pw}$ is assumed to be 1‰ (i.e., carbon isotope composition of host rock). $\delta^{13}C_{HCO_3^-}^{CH4}$ can be calculated from equations 3 and 4. Namely, assuming $\delta^{13}C$ for $CH_3SH$ and $CH_3SCH_3$ ($\delta^{13}C_{MS}$) at −30‰, and fractionation during methanogenesis at −60‰[23] (i.e., $\delta^{13}C_{CH4} - \delta^{13}C_{MS} = -60‰$, or $\delta^{13}C_{CH4}$ = −90‰), $\delta^{13}C_{HCO_3^-}^{CH4}$ should be +150‰. Thus, the isotopic difference between MT calcspar and host rock (similar to $\delta^{13}C_{HCO_3^-}^{pw}$) should be

$$\delta^{13}C_{MT} - \delta^{13}C_{HR} = f_{CH4} \times (\delta^{13}C_{HCO_3^-}^{CH4} - \delta^{13}C_{HCO_3^-}^{pw}) \quad \text{(S-10)}$$

If MSR-derived $HCO_3^-$ involves in MT microspar precipitation, equations S-9 and S-10 can be written as:

$$\delta^{13}C_{MT} = f_{CH4} \times \delta^{13}C_{HCO_3^-}^{CH4} + f_{MSR} \times \delta^{13}C_{HCO_3^-}^{MSR} + (1 - f_{CH4} - f_{MSR}) \times \delta^{13}C_{HCO_3^-}^{pw} \quad \text{(S-11)}$$

$$\delta^{13}C_{MT} - \delta^{13}C_{HR} = f_{CH4} \times (\delta^{13}C_{HCO_3^-}^{CH4} - \delta^{13}C_{HCO_3^-}^{pw}) + f_{MSR} \times (\delta^{13}C_{HCO_3^-}^{MSR} - \delta^{13}C_{HCO_3^-}^{pw})$$

(S-12)

Where $\delta^{13}C_{HCO_3^-}^{MSR}$ and $f_{MSR}$ are the isotopic composition and fraction of MSR-derived $HCO_3^-$.

**Supplementary References**


1 Liu, Y., Cai, G., Kuang, H., Meng, X. & GE, M. The discovery of paleo-weathering crusts between Sinian W anlong Formation and Badaojiang Formation in southern Jilin Provinc. *Acta Petrologica et Mineralogica* **24**, 47-52, (2005).

2 Canfield, D. E. Sulfate reduction in deep-sea sediments. *American Journal of Science* **291**, 177-188, (1991).

3 Habicht, K. S. & Canfield, D. E. Sulfur isotope fractionation during bacterial sulfate reduction in organic-rich sediments. *Geochimica et Cosmochimica Acta* **61**, 5351-5361, (1997).

4 Pingitore, N. E., Jr., Meitzner, G. & Love, K. M. Identification of sulfate in natural carbonates by x-ray absorption spectroscopy. *Geochimica et Cosmochimica Acta* **59**, 2477-2483, (1995).

5 Kampschulte, A., Bruckschen, P. & Strauss, H. The sulphur isotopic composition of trace sulphates in Carboniferous brachiopods: implications for coeval seawater, correlation with other geochemical cycles and isotope stratigraphy. *Chemical Geology* **205**, 149-173, (2001).

6 Kampschulte, A. & Strauss, H. The sulfur isotopic evolution of Phanerozoic seawater based on the analysis of structurally substituted sulfate in carbonates. *Chemical Geology* **204**, 255-286, (2004).

7 Galy, A., Bar-Matthews, M., Halicz, L. & O'Nions, R. K. Mg isotopic composition of carbonate: insight from speleothem formation. *Earth and Planetary Science Letters* **201**, 105-115, (2002).

8 Young, E. D. & Galy, A. The isotope geochemistry and cosmochemistry of magnesium. *Reviews in Mineralogy & Geochemistry* **55**, 197-230, (2004).

9 Fantle, M. S. & Higgins, J. The effects of diagenesis and dolomitization on Ca and Mg isotopes in marine platform carbonates: Implications for the geochemical cycles of Ca and Mg. *Geochimica et Cosmochimica Acta* **142**, 458-481, (2014).

10 Higgins, J. A. & Schrag, D. P. Constraining magnesium cycling in marine sediments using magnesium isotopes. *Geochimica et Cosmochimica Acta* **74**, 5039-5053, (2010).

11 Higgins, J. A. & Schrag, D. P. Records of Neogene seawater chemistry and diagenesis in deep-sea carbonate sediments and pore fluids. *Earth and Planetary Science Letters* **357–358**, 386-396, (2012).

12 Higgins, J. A. & Schrag, D. P. The Mg isotopic composition of Cenozoic seawater – evidence for a link between Mg-clays, seawater Mg/Ca, and climate. *Earth and Planetary Science Letters* **416**, 73-81, (2015).

13 Immenhauser, A. *et al.* Magnesium-isotope fractionation during low-Mg calcite precipitation in a limestone cave - field study and experiments. *Geochimica et Cosmochimica Acta* **74**, 4346-4364, (2010).

14 Wang, Z. *et al.* Experimental calibration of Mg isotope fractionation between aragonite and seawater. *Geochimica et Cosmochimica Acta* **102**, 113-123, (2013).

15 Li, W., Chakraborty, S., Beard, B. L., Romanek, C. S. & Johnson, C. M. Magnesium isotope fractionation during precipitation of inorganic calcite under laboratory conditions. *Earth and Planetary Science Letters* **333–334**, 304-316, (2012).

16 Saulnier, S., Rollion-Bard, C., Vigier, N. & Chaussidon, M. Mg isotope fractionation during calcite precipitation: An experimental study. *Geochimica et Cosmochimica Acta* **91**, 75-91, (2012).

17 Huang, K.-J. *et al.* Magnesium isotopic compositions of the Mesoproterozoic dolostones:

Implications for Mg isotopic systematics of marine carbonate. *Geochimica et Cosmochimica Acta* **164**, 333-351, (2015).

18 Li, W., Beard, B. L., Li, C., Xu, H. & Johnson, C. M. Experimental calibration of Mg isotope fractionation between dolomite and aqueous solution and its geological implications. *Geochimica et Cosmochimica Acta* **157**, 164-181, (2015).

19 Galy, A. *et al.* Magnesium isotope heterogeneity of the isotopic standard SRM980 and new reference materials for magnesium-isotope-ratio measurements. *Journal of Analytical Atomic Spectrometry* **18**, 1352-1356, (2003).

20 Tucker, M. E. & Wright, V. P. *Carbonate Sedimentology*. (Blackwell Scientific, 1990).

21 Hardie, L. A. Secular variation in seawater chemistry: An explanation for the coupled secular variation in the mineralogies of marine limestones and potash evaporites over the past 600 my. *Geology* **24**, 279-283, (1996).

22 Pollock, M. D., Kah, L. C. & Bartley, J. K. Morphology of molar-tooth structures in Precambrian carbonates: influence of substrate rheology and implications for genesis *Journal of Sedimentary Research* **76**, 310-323, (2006).

23 Summons, R. E., Franzmann, P. D. & Nichols, P. D. Carbon isotopic fractionation associated with methylotrophic methanogenesis. *Organic Geochemistry* **28**, 465-475, (1998).
